# The relationship between Aldosterone level and various LV conditions in patients with End-stage renal disease

**DOI:** 10.22088/cjim.10.1.36

**Published:** 2019

**Authors:** Vida Nesarhosseini, Hossein Mohsenipouya, Atieh Makhlough, Rozita Jalalian

**Affiliations:** 1 Department of Cardiology, Cardiovascular Research Center, Mazandaran University of Medical Sciences, Sari, Iran; 2 Cardiovascular Research Center, Mazandaran University of Medical Sciences, Sari, Iran; 3 Department of Nephrology, Cardiovascular Research Center, Mazandaran University of Medical Sciences, Sari, Iran

**Keywords:** Aldosterone, Left ventricular hypertrophy, ESRD

## Abstract

**Background:**

Aldosterone has been assumed to be implicated in left ventricular hypertrophy (LVH). Preventing the progression of LVH in the early period of end-stage renal disease (ESRD) can increase patient survival. In this study, therefore, we analyzed the relationship between aldosterone level and LVH in ESRD patients who underwent hemodialysis at Fatemeh Zahra Hospital and Imam Khomeini Hospital in Sari, Iran from 2016 to 2017.

**Methods:**

This research is a case-control study involving 69 patients, divided into the case group (n=52, exhibiting LVH) and the control group (n=17, no ventricular hypertrophy observed in the echocardiography). The relationship between the patients’ serum aldosterone levels and

LVH was evaluated on the basis of relative wall thickness (RWT).

**Results:**

Among the patients, 20.3% had normal cardiac conditions, 53.6% eccentric hypertrophy (EH), 4.3% exhibited concentric remodeling (CR), and 21.7% had concentric hypertrophy (CH). In other words, 24.6% of the patients belonged to the control group, and 75.4% belonged to the case group. The results indicated a significant difference (P=0.006) in average aldosterone levels between the case (165.11±80.8) and control (115.76±72.47) groups and a significant difference in aldosterone levels among the four subgroups (P=0.03), with the levels of the CH group being higher than those of the EH group.

**Conclusion:**

Based on the results of the study, a significant relationship exists between plasma aldosterone level and LVH in ESRD patients. Serum aldosterone level is therefore a predictor of LVH.

Cardiovascular diseases are major causes of mortality among patients with end-stage renal disease (ESRD) (1, 2), who are highly at risk of developing left ventricular hypertrophy (LVH) (3, 4). Cardiovascular disease includes coronary artery diseases (CAD) such as angina and myocardial infarction (commonly known as a heart attack). Most cardiovascular diseases affect older adults. In the United States, 11% of people between 20 and 40 have CVD, while 37% between 40 and 60, 71% of people between 60 and 80, and 85% of people over 80 have CVD (5). LVH is a condition in which muscular hypertrophy occurs as a result of the difficulty of the heart’s left ventricle in pumping blood. LVH causes systolic and diastolic dysfunction, reduces blood supply in coronary arteries, and eventually gives rise to side effects such as ischemia, myocardial infarction, heart failure, and sudden death.

The physiological factors responsible for LVH are isometric exercise, pregnancy, old age, obesity, and salt intake; the pathological factors implicated in the disease are relevant to hypertension (HTN), aortic stenosis (AS), aortic insufficiency, hypertrophic cardiomyopathy, and other cardiomyopathies (6). The specific incidence rate of LVH among patients with ESRD is 68% to 89%, and the disease often occurs in progressive form (3, 7). The prevention of LVH can improve the survival of ESRD patients. For example, a 10% reduction in LVH results in a 28% decrease in the risk of cardiac mortality among patients on dialysis (8, 9). LVH is also a powerful predictor of mortality and an independent risk factor for patients with chronic kidney disease (CKD) (10-12), which involves a range of pathological mechanisms concerned with abnormal kidney function and progressive reduction in glomerular filtration. CKD is most commonly caused by diabetic nephropathy, diabetes mellitus type 2, and hypertensive nephropathy. Its diagnosis can be considerably facilitated by laboratory tests, imaging studies, frequent measurements of plasma urea and creatinine concentrations, and renal biopsy. In imaging studies, observations of metabolic bone disease with hyperphosphatemia, hypoxia, increased levels of parathyroid hormone (PTH) and radiological disorders, normocytic and normochromic anemia, and reduced kidney size (<8.5 cm) strongly favor CKD (6).

ESRD is the final stage of CKD, and the genesis of hypertrophy in the former may be caused by several factors, such as HTN (13), increased PTH levels (14), and the renin–angiotensin–aldosterone system (11). Clinical evidence indicated that aldosterone plays a pathologic role in cardiac hypertrophy—a role that appears to be independent of its effect on blood pressure (11, 15-17). Aldosterone is a *mineralocorticoid* that is produced in adrenal cortical glomeruli and plays a substantial role in sodium and water reabsorption as well as potassium secretion (6). Primary aldosteronism appears to increase the incidence of LVH, as evidenced by the higher number of LVH cases in patients with primary aldosteronism than in subjects with renovascular HTN (18). Ritz and Amman found that comparing with patients who have preserved renal function, ESRD patients with LVH exhibit myocyte hypertrophy, loss of myocardial capillaries, increased interstitial fibrosis, and reduced capillary-to-myocyte ratio (19). The morphological findings of in vitro and in vivo studies showed that increased levels of aldosterone affect non-myocyte cells (endothelial and cardiac fibroblasts) and accordingly result in collagen accumulation and myocardial fibrosis, which can contribute to LVH pathogenesis (17). Some studies found that plasma aldosterone levels are related to concentric LVH (17, 20). During treatment of concentric LVH in certain studies, for example, aldosterone inhibitors reduced left ventricular mass index (LVMI) in patients with HTN but did not eliminate LVH (21, 22). Other studies suggested that in ESRD patients on dialysis, aldosterone inhibition causes LVH to regress (23). In research involving ESRD patients of different races, however, no relationship was observed between aldosterone level and LVH (24, 25). As seen in the discussion above, the role of aldosterone in hypertrophy incidence is known, with studies providing contradictory results in this regard (26-28). The necessity of preventing LVH and implementing timely intervention during the early stages of ESRD (8, 16, 22) highlights the importance of exploring whether decreased aldosterone levels in ESRD patients reduce the incidence of LVH. Accordingly, the present research was conducted with the aim of analyzing the relationship between aldosterone level and LVH in ESRD patients undergoing dialysis at Imam Khomeini Hospital and Fatemeh Zahra Hospital in Sari, Iran from 2016 to 2017.

## Methods

**Study design and patients: **This case-control study involved 69 ESRD patients admitted into Fatemeh Zahra Hospital and Imam Khomeini Hospital from 2016 to 2017. Sixty nine participants were selected via census sampling, 52 patients were in the case group who had LVH, and 17 patients in the control group with no hypertrophy. Data were collected from the field using a questionnaire validated by six cardiology specialists. The inclusion criteria were as follows: being on dialysis twice a week for at least three months, having an ejection fraction (EF) greater than 40%, and having a blood pressure lower than 160/100. The exclusion criteria were: having a blood pressure greater than 160/100; the presence of coronary artery disease, cardiac valve disease, atrial fibrillation, congestive heart failure, diabetes mellitus, and having taken angiotensin-converting enzyme inhibitors (ACEIs) and angiotensin-receptor blockers (ARBs) during the past two weeks. After the patients were informed of the necessity of the study and sampling, written consent was obtained from them. All the eligible patients underwent B-Mode 2D echocardiography supervised by a cardiologist and were then divided into the case group, composed of patients exhibiting LVH, and the control group, consisting of patients whose echocardiograms reflected no LVH.

**Measures: ** Intervening factors such as potassium and blood glucose levels, blood pressure, period of hemodialysis, age, and sex were homogenized. In both groups, biochemical factors such as plasma aldosterone, sodium, potassium, creatinine, urea, triglyceride, high-density lipoprotein, and low-density lipoprotein levels were measured. The systolic and diastolic blood pressure and body mass index of the patients were recorded in the questionnaire. Enzyme-linked immunosorbent assay (IBL, Germany) was conducted to measure the aldosterone levels of the patients. Two weeks after the primary examination, a blood glucose test to exclude diabetic patients from studying and matching patients and echocardiography were conducted. In hypertensive patients, the ACEI and ARB drugs were substituted with other medications for two weeks. This issue has been informed to patients.

Fasting blood sugar was measured after 20 minutes of rest in a supine position. The LV diastolic dimension (LVDd), interventricular septal thickness (IVST), posterior wall thickness (PWT), and LV ejection fraction (LVEF) were measured in the parasternal long axis view using 2D echocardiography (Philips). The body area for LV mass measurement was calculated using the Devereaux formula, and the value was recorded as the LV mass index. The patients having an LVMI greater than 125 g/m^2^ were placed in the case group. Relative wall thickness (RWT) was calculated by multiplying the PWT value by 2 and then dividing the result by the LVDd value. Values higher than 0.44 were considered to reflect increased RWT. On the basis of the geometric values of the left ventricle, the patients were further subdivided into four groups, namely, the normal (N) group, who exhibited normal RWT and LVMI; the concentric remodeling (CR) group, who presented normal LVMI and increased RWT; the eccentric hypertrophy (EH) group, comprising patients with increased LVMI and normal RWT; and the concentric hypertrophy (CH) group, consisting of patients with increased LVMI and RWT. The patients’ blood pressure and heartbeat were measured in a sitting position (after 5 minutes of rest).

**Ethical considerations:** The study was conducted in accordance with the principles of the Declaration of Helsinki and informed consent was obtained from all patients.

**Statistical methods:** To evaluate whether the data were normally distributed, the Kolmogorov–Smirnov test was performed. Given the non-normal distribution, non-parametric tests were used to analyze the data. All the data were analyzed using the Statistical Package for the Social Sciences Version 22. Kruskal–Wallis and Mann–Whitney tests were carried out to determine the differences in LVH levels between the groups. A p-value less than 0.05 was considered statistically significant.

## Results

The patients’ ages ranged from 35 to 91 years, with the average being 62±13.86. Out of the 69 patients, 34 (49.3%) were women and 35 (50.7%) were men. The total average sodium and potassium levels and systolic and diastolic blood pressure were 142.29±2.98 and 4.54±0.72 and 126.53±10.42 and 77.20±10.17, respectively. These values were homogenized in the two groups (p>0.05, table 1).

**Table 1:** Frequency distribution of clinical variables

**Variables **	**Control**	**Case**	**Total**	**P-value**
	**Mean±SD**	**Mean±SD**	**Mean±SD**	
Age	59.31±15.30	62.62±13.38	61.78±13.86	0.351
Sodium	141.36±2.77	142.6±3.01	142.29±2.98	0.783
Potassium	4.5±0.63	4.56±0.75	4.54±0.72	0.622
SBP	123.68±10.65	127.50±10.26	126.53±10.42	0.311
DBP	76.84±9.45	77.32±10.48	77.20±10.17	0.636

Out of the 69 patients, 14 (20.3%) belonged to the N group, 37 (53.6%) the EH group, 3 (4.3%) the CR group, and 15 (21.7%) belonged to the CH group (table 2).

**Table 2:** Frequency Distribution Based on Geometric Values of the Left Ventricle

**Group**	**Frequency (percent)**	**Frequency among women**	**Frequency among men**
N	14 (20.3%)	9	5
EH	37 (53.6%)	16	21
CR	3 (4.3%)	3	0
CH	15 (21.7%)	6	9
Total	69 (100%)	34	35

(N) Normal group= normal values of RWT and LVMI, (CR) concentric remodeling group= normal LVMI and increased RWT, (EH) Eccentric hypertrophy group= increased LVMI and normal RWT; (CH) concentric hypertrophy group= increased LVMI and increased RWT

 With respect to primary grouping, 17 patients (24.6%) belonged to the control group, whose subgroups were the N and CR groups; 52 (75.4%) belonged to the case group, whose subgroups comprised the CH and EH patients. The results of the Mann–Whitney test indicated a significant difference in aldosterone levels between the case group (165.11±80.8) and the controls (115.76±72.47, P=0.006). Table 3 shows the average aldosterone level of each of the four subgroups. The average aldosterone level of all the 69 participants was 152.95±81.25. The results of the Kruskal–Wallis test indicated a significant statistical difference in aldosterone levels among the four subgroups (P=0.032).

**Table 3:** Average aldosterone levels of the subgroups

**Groups**	**Aldosterone Level**	**P-value**
	**Mean** **±** **SD**	
N	116.14±72.21	^*^P=0.032
EH	154.21±76.38	
CR	114±90	
CH	192±87.9	
Total	152.95±81.25	

(N) Normal group= normal values of RWT and LVMI, (CR) concentric remodeling group= normal LVMI and increased RWT, (EH) Eccentric hypertrophy group= increased LVMI and normal RWT; (CH) concentric hypertrophy group= increased LVMI and increased RWT

* Kruskalwallis test

## Discussion

Patients with CKD exhibit abnormally high aldosterone levels (29). Although the relationship between LV mass and aldosterone levels in hypertensive and diabetic patients was well clarified, this issue has not been examined with respect to CKD patients, especially those undergoing dialysis (11). To address this gap, this study was conducted with the aim of evaluating the relationship between aldosterone level and LVH in ESRD patients on dialysis. The findings indicated a significant relationship between high aldosterone levels and increased hypertrophy of the left ventricle. This outcome, in turn, can increase the mortality of ESRD patients on dialysis, the mortality resulting from cerebrovascular diseases, and the mortality and morbidity of cardiovascular diseases (30-33). Other studies presented similar results (11, 15, 24, 34). Bomback et al. (29), for instance, found that the average aldosterone levels of ESRD patients were higher than those of healthy individuals (26.7 and 12.4 mg/dl, respectively). The difference was significant. In the current work, the aldosterone levels of the case and control groups were 165.11±80.8 and 115.76±72.47, respectively, also exhibiting a significant difference.

The mean aldosterone level in the whole patient is 152.95±81.25 and the normal level in aldosterone is given in table 3. In our study, about 75% of the patients suffered from LVH. In this proportion of patients, CH was more significantly related to aldosterone level than EH. Mule et al. (2015), who examined the concentration of plasma aldosterone and its relationship with LVM in CKD hypertensive patients, indicated that LVM, RWT, and plasma aldosterone concentration increased in these patients—conditions that are associated with the early stages of renal failure. The authors also reported a close relationship between plasma aldosterone concentration and concentric LVH, compared with patients with eccentric LVH, those suffering from eccentric LVH exhibited lower plasma aldosterone concentrations (22). Nakahar et al. (20) investigated 57 patients with CH and EH and found a significant relationship among plasma aldosterone level, LVMI, and the wall thickness of the left ventricle. This result was particularly prominent in the concentric group, consistent with our findings. Taken together, these findings indicated that eccentric LVH patients, who are mostly people with volume overload, exhibit increased LVMI but normal RWT.

 In patients with concentric LVH, who are mostly people afflicted with blood pressure or pressure overload, LVMI and RWT increase; in the latter type of patients, blood aldosterone levels also significantly increased. In the studies conducted by Adebiyi et al (25), El-Gharbawy et al. (35), and Vasan (28), however, no significant relationship was found between LVMI and plasma aldosterone. The difference in findings can be attributed to type of population studied.

In conclusion,** o**n the whole, the results and their comparison with those of other studies indicated a significant relationship between plasma aldosterone level and LVH in ESRD patients. Therefore, monitoring serum aldosterone levels can help predict the occurrence of LVH.

**Power and limitation study**

This study can be used as a marker for predicting hypertrophy. But regarding the limitation of the study, participants were often excluded from the study due to underlying illness and the use of ARB, ACE medications.
